# CAREGIVER STRAIN AMONG AFRICAN AMERICAN AND HISPANIC MALE CAREGIVERS WITH CHRONIC CONDITIONS

**DOI:** 10.1093/geroni/igac059.398

**Published:** 2022-12-20

**Authors:** Matthew Smith, Chung Lin Kew, Tiffany Washington, Caroline Bergeron, Ashley Merianos, Ledric Sherman, Kirby Goidel

**Affiliations:** Texas A&M University, College Station, Texas, United States; Texas A&M University, College Station, Texas, United States; University of Georgia, Athens, Georgia, United States; University of Ottawa, Ottawa, Ontario, Canada; University of Cincinnati, Cincinnati, Ohio, United States; Texas A&M University, College Station, Texas, United States; Texas A&M University, College Station, Texas, United States

## Abstract

Caregiving strain often stems from caregivers’ unmet needs and is a risk factor for physical and psychological ill-health. This study aims to identify factors associated with caregiver strain among middle-aged and older African American and Hispanic male caregivers living with one or more chronic conditions. Data were collected from 431 male caregivers using a web-based survey (55% African American, 45% Hispanic). Linear regression models were fitted to assess factors associated with caregiver strain, which was measured using caregiving difficulty items from Behavioral Risk Factor Surveillance System. On average, participants were age 54.9(±9.51) years, they self-reported chronic conditions were 3.74(±2.62), and their caregiver strain was 14.7(±7.30). Among African American caregivers, higher caregiver strain was positively associated with living with children below age 18 (β=0.14, P=0.045) and feelings of social disconnectedness (β=0.16, P=0.018) and depression (β=0.15, P=0.035). Conversely, caregiver strain was negatively associated with having insurance coverage (β=-1.34, P=0.028) and disease self-management efficacy (β=-2.26, P=< 0.001. Among Hispanic caregivers, higher caregiver strain was negatively associated with age (β=-0.28, P=< 0.001) and positively associated with feelings of social disconnectedness (β=0.16, P=0.041). Findings suggest African American and Hispanic males with chronic conditions have differing caregiving experiences. Compared to Hispanic men, contributors to caregiving strain among African American men were multifaceted and associated with financial resources, household dynamics, mental health, and the ability to self-manage their chronic conditions. While bolstering social connectedness may offset caregiver strain, tailored mental health and disease-management programming are needed to meet the specific needs of African American and Hispanic male caregivers.

